# Localized hydroxyl radical generation at mmol/L and mol/L levels in water by photon irradiation

**DOI:** 10.3164/jcbn.18-15

**Published:** 2018-04-03

**Authors:** Yukihiro Ogawa, Emiko Sekine-Suzuki, Megumi Ueno, Ikuo Nakanishi, Ken-ichiro Matsumoto

**Affiliations:** 1 Graduate School of Advanced Integration Science, Chiba University, 1-33 Yayoicho, Inage-ku, Chiba-shi, Chiba 263-8522, Japan; 2 Quantitative RedOx Sensing Team, Department of Basic Medical Sciences for Radiation Damages, National Institute of Radiological Sciences, National Institutes for Quantum and Radiological Science and Technology, 4-9-1 Anagawa, Inage-ku, Chiba-shi, Chiba 263-8555, Japan; 3 Graduate School of Science, Chiba University, 1-33 Yayoicho, Inage-ku, Chiba-shi, Chiba 263-8522, Japan

**Keywords:** reactive oxygen species, X-ray, γ-ray, electron paramagnetic resonance spin-trapping technique, molecular density

## Abstract

The generation of localized hydroxyl radical (^•^OH) in aqueous samples by low linear energy transfer irradiation was investigated. Several concentrations of 5,5-dimethyl-1-pyrroline-*N*-oxid solution (from 0.5 to 1,680 mmol/L) were prepared and irradiated with an identical dose of X-ray or γ-ray. The density of ^•^OH generation in aqueous solution was evaluated by the electron paramagnetic resonance spin-trapping technique using 5,5-dimethyl-1-pyrroline-*N*-oxid as an electron paramagnetic resonance spin-trapping agent. The relationship between the molecular density of 5,5-dimethyl-1-pyrroline-*N*-oxid in the samples and the concentration of 5,5-dimethyl-1-pyrroline-*N*-oxid-OH generated in the irradiated samples was analyzed. Two different characteristic linear trends were observed in the 5,5-dimethyl-1-pyrroline-*N*-oxid-OH/5,5-dimethyl-1-pyrroline-*N*-oxid plots, which suggested ^•^OH generation in two fashions, i.e., mmol/L- and mol/L-level local concentrations. The dose, dose rate, and/or the energy of photon irradiation did not affect the shapes of the 5,5-dimethyl-1-pyrroline-*N*-oxid-OH/5,5-dimethyl-1-pyrroline-*N*-oxid plots. Moreover, the addition of 5 mmol/L caffeine could cancel the contribution of mmol/L-level ^•^OH generation, leaving a trace of mol/L-level ^•^OH generation. Thus, the localized mmol/L- and mol/L-level generations of ^•^OH, which were independent of experimental parameters such as dose, dose rate, and/or the energy of photon of low linear energy transfer radiation, were established.

## Introduction

Water (H_2_O) is the most abundant molecule in living organisms. The indirect action of radiation, which causes biomolecular damages through reactive species generated by radiolysis of H_2_O,^(1)^ might be a major factor of radio-biological effects.^(2,3)^ Reactive oxygen species (ROS), such as hydroxyl radicals (^•^OH), superoxide anion (O_2_^•−^), and hydrogen peroxide (H_2_O_2_), are the main reactive species that can mediate radio-biological effects. Among the radiation-induced ROS, the ^•^OH is recognized as an initial product and an important player in biological effects owing to its high reactivity. For low linear energy transfer (LET) radiation, such as X-ray and γ-ray, the indirect action caused by water radiolysis products contributes to DNA damages.^(4–7)^ Therefore, quantification and elimination of ROS induced by radiation are important for radioprotection.

^•^OH can be detected using the electron paramagnetic resonance (EPR) spin-trapping method. Spin-trapping agents can react with short-lived free radicals, such as ^•^OH, and detect them as a relatively stable nitroxyl radical form, which is called a spin adduct.^(8–10)^ 5,5-Dimethyl-1-pyrroline-*N*-oxide (DMPO) is probably the most common spin-trapping agent.^(10–16)^ In a previous report, two different localized ^•^OH, i.e., “sparse” and “dense”, generated by irradiation of aqueous samples with ionizing radiation were detected using the EPR spin-trapping technique.^(15)^ It was reported that the ratio of sparse and dense ^•^OH generation varied according to LET, with sparse ^•^OH generation decreasing with increasing LET.

When ionizing radiation is passed through an aqueous sample, such as cells, radiation-induced ionization and/or excitation of H_2_O molecules occur. Sequentially, a cluster of primary reactive species is produced in the dense ionization area, which is a few nm in diameter, called “spur.”^(4)^ Some of the ^•^OH generated in the spur may interact with each other to produce secondary or later generated ROS, which then diffuse into the bulk solution. For the reaction of ^•^OH among themselves before their lapse, the distance between two neighboring ^•^OH may have to be less than 2 nm, which corresponds to a concentration of 200 mmol/L or more. Such a dense ^•^OH generation must occur in the spur; however, the occurrence of dense ^•^OH generation is not sufficiently experimentally clear. This study focused on low LET radiation-induced ^•^OH generation, and attempted to prove extremely dense ^•^OH generation in spur volume.

## Materials and Methods

### Chemicals

5,5-Dimethyl-1-pyrroline-*N*-oxide (DMPO) was purchased from Dojindo Laboratories, Ltd. (Kumamoto, Japan). 4-Hydroxy-2,2,6,6-tetramethylpiperidin-1-oxyl (TEMPOL) and caffeine were purchased from Sigma-Aldrich (St. Louis, MO). Deionized water (deionization by the Milli-Q system, Merck Millipore, Billerica, MA) was used for all sample preparations.

### Preparation of DMPO Solution

Aqueous solutions of DMPO were freshly prepared just before irradiation according to previous reports.^(15,16)^ The concentrations of DMPO were so planned that the molecular distance of DMPO dissolved in water would be some arbitrary distance from 1 nm to 15 nm. Considering the volume occupied by a single DMPO molecule to be a cube, the length of the side of the cube was defined as “probe-to-probe distance,” and the reciprocal of “probe-to-probe distance” was defined as “probe density,” i.e., the number of ^•^OH probe on the unit distance. The relation between concentration, probe-to-probe distance, and probe density of DMPO is shown in Table 1. An aliquot (200 µl) of reaction mixture was transferred into a polyethylene microtube (1.5 ml volume) and kept on ice until irradiation.

### X-ray Irradiation

X-ray irradiations were performed using a PANTAK 320 X-ray generator (Shimadzu, Kyoto, Japan) with an effective energy of 80 keV under an X-ray tube voltage of 200 kV, X-ray tube current of 20 mA, and the pre-filter materials of copper and aluminum with thicknesses of 0.5 mm. Experiments were performed with 3.2, 4.8, or 6.4 Gy/min, when the distance between the X-ray tube and the sample were 300, 232, or 212 mm, respectively. The samples were irradiated with 32 Gy or 16 Gy.

### γ-Ray Irradiation

γ-Ray irradiations were performed using a ^137^Cs γ-ray source irradiator (Gammator M, Radiation Machinery, Parsippany, NJ). The disintegration rate of the ^137^Cs source was 49.4 TBq. The dose rate was 7.9 Gy/min when the distance between the ^137^Cs γ-source and the sample was 3.81 cm (1.5 inch). The samples were irradiated with 32 Gy for an irradiation time of 4 min.

### Canceling mmol/L-level ^•^OH by Caffeine

Caffeine, which has a high ^•^OH scavenging activity,^(16)^ was added to the series of DMPO solutions listed in Table 1. Several values were reported as the rate constants of caffeine with ^•^OH, e.g., 5.9, 6.9 or 7.3 × 10^9^ M^−1^ s^−1^.^(17–19)^ The final concentration of caffeine was 5 mmol/L. An aliquot (200 µl) of reaction mixture was identically treated, and the samples were then irradiated by X-ray as described above.

### EPR Measurement

An aliquot (100 µl) of the irradiated aqueous solution of DMPO was drawn into a PTFE tube (i.d.: 0.32 ± 0.001 in, wall thickness: 0.002 ± 0.0005 in; ZEUS, Orangeburg, SC), placed into a special quartz sample tube fixed in the transverse electric (TE) mode cavity, and measured using an X-band EPR spectrometer (JES-TE100, JEOL, Tokyo, Japan) equipped with a WIN-RAD EPR Data Analyzer System (Radical Research, Inc., Hino, Tokyo).

To avoid the contribution of overlapping DMPO-H (^•^H adduct of DMPO) spectra, the second line from the lower end of the magnetic field of the DMPO-OH spectrum, which comprises the characteristic 1:2:2:1 four-line, was recorded under the following conditions: microwave frequency: 9.45 GHz, microwave power: 2 mW, lower magnetic field: 336.1 mT, field sweep width: 2.5 mT, field sweep resolution: 1,024 points, sweep time: 60 s, time constant: 0.01 s, field modulation frequency: 100 kHz, and field modulation width: 0.063 mT. EPR measurements were started approximately 2 min after irradiation, and were repeated every 1 min for 10 min. The time course of DMPO-OH signal was obtained for each sample. The acquired EPR spectra (1,024 point digital data) were analyzed using an in-house line fitting program. A Gaussian line shape was fitted on the experimental spectrum, and signal height and line width of the fitted Gaussian line were recorded. The EPR signal intensity was evaluated as follows: (signal height) × (line width)^2^. The concentration of DMPO-OH at the end of X-ray irradiation (time = 0) was obtained by extrapolating the decay curve of DMPO-OH to time = 0. Finally, the total amount of DMPO-OH during irradiation was obtained according to a previously described method.^(16)^ The concentration of DMPO-OH was calculated based on the EPR signal intensities of the accurately prepared 0.2 mmol/L TEMPOL aqueous solutions, which were measured with the same geometry and EPR parameters as mentioned above.

## Results

**Fig. 1:**
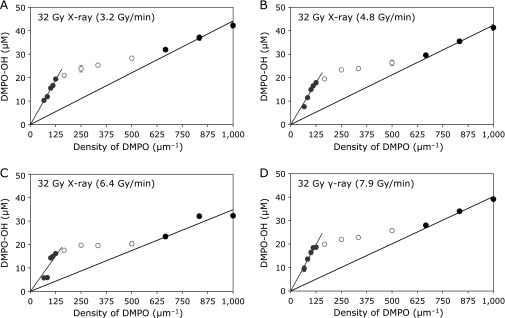
Effect of dose rate and photon type on density of radiation-induced ^•^OH generation. The DMPO-OH concentration generated by an identical irradiation dose to a series of DMPO reaction mixtures was plotted against DMPO density in the reaction mixture: (A) 32 Gy X-ray (3.2 Gy/min), (B) 32 Gy X-ray (4.8 Gy/min), (C) 32 Gy X-ray (6.4 Gy/min), and (D) 32 Gy γ-ray from a ^137^Cs source (7.9 Gy/min) irradiations. The marks and error bars indicate mean ± SD of four samples. The lines through the origin are the least squares linear approximation calculated for gray and black solid circles of the plots.

The characteristic three-phase profiles shown in Fig. 1 indicate the relationship between the density of DMPO and the concentration of DMPO-OH generated in the reaction mixtures irradiated by an identical dose. The same pattern was observed in a previous report.^(15)^ When the density of DMPO as a ^•^OH detector was insufficient to the local density of the generated ^•^OH, the liner plots that pass through the origin would be observed by increasing of the DMPO density. When the density of DMPO in the reaction system was sufficiently excessive to the local density of the generated ^•^OH, the plots of DMPO-OH concentration reached a plateau. A linear increase in DMPO-OH concentration associated with an increase in DMPO density was observed for lower DMPO densities (<125 µm^−1^). An articulated inflection point could be observed at 125 µm^−1^ of DMPO density, which corresponds to 3.2 mmol/L of DMPO concentration. The plot of DMPO-OH for a DMPO density higher than 667 µm^−1^ (DMPO concentration >492 mmol/L) showed another linear trend that passed through the origin but did not show an inflection point till 1,000 µm^−1^ DMPO density (1.7 mol/L concentration). The same trend, i.e., an inflection point and two linear profiles that pass through the origin, was observed at the same position even though X-ray irradiations were performed at different dose rates (Fig. 1A–C). The distortion in the plot in Fig. 1C is probably because of the very small distance between the sample and the X-ray tube. Even for γ-ray irradiation, which had a different low LET photon radiation source, the same three-phase trend was observed (Fig. 1D).

**Fig. 2:**
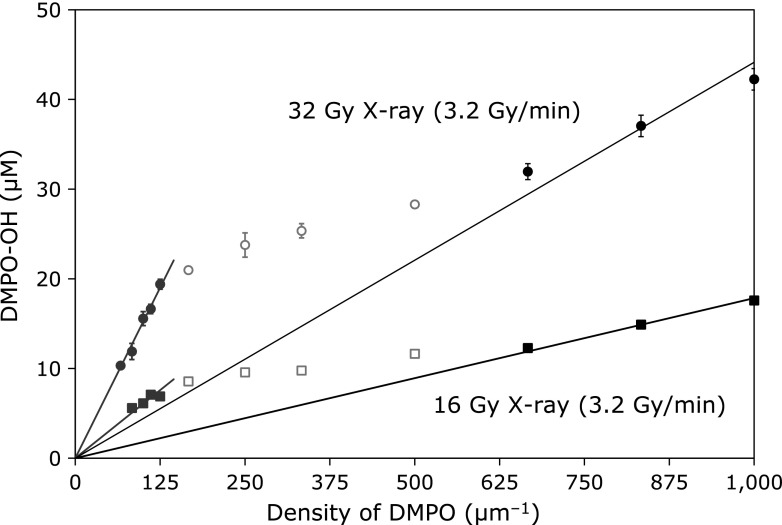
Effect of radiation dose on density of radiation-induced ^•^OH generation. The DMPO-OH concentration generated by an identical irradiation dose to a series of DMPO reaction mixtures were plotted against DMPO density in the reaction mixture. The squares indicate result obtained by 16 Gy X-ray (*n*≥4). The circles are re-plots of Fig. 1A as the reference. The lines through the origin are the least squares approximation calculated for gray and black solid marks.

Figure 2 shows the profiles when the total dose was reduced from 32 Gy to 16 Gy. The dose rate of 3.2 Gy/min was the same as in previous experiments shown in Fig. 1A. The amount of generated DMPO-OH reduced to half when the total dose was halved. The total dose did not affect the position of the inflection point in the DMPO density plots. Moreover, two linear profiles that passed through the origin were obtained at the same position with half-dose irradiation.

**Fig. 3:**
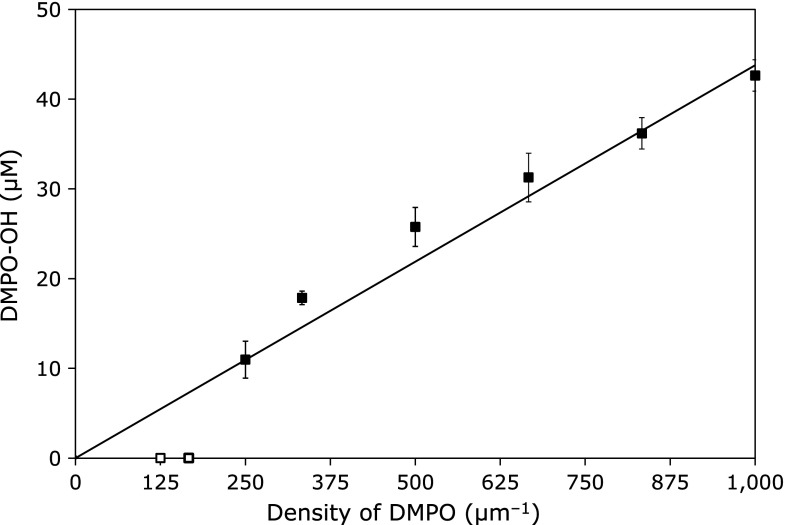
Effect of ^•^OH scavenger on density of radiation-induced ^•^OH generation. The DMPO-OH concentration generated by an identical irradiation dose to a series of DMPO reaction mixtures was plotted against DMPO density in the reaction mixture when 5 mmol/L caffeine was added to the samples. The marks and error bars indicate mean ± SD of four samples. The line through the origin is the least squares approximation calculated for the plots.

Figure 3 shows the profile when 5 mmol/L caffeine was added to the reaction system as a radical scavenger. DMPO-OH was not detected when the DMPO density was less than 167 µm^−1^ (7.7 mmol/L). On the other hand, a single linear increase in DMPO-OH concentration was obtained with increasing DMPO density. This liner trend in Fig. 3 corresponds to the liner trends obtained in Fig. 1 and 2 at higher DMPO densities.

## Discussion

As shown in the longitudinal axis of Fig. 1, the measured concentration of DMPO-OH in the irradiated reaction mixture was in µmol/L level. This is the average concentration of DMPO-OH in a certain volume of sample. The concentration of DMPO-OH measured at the inflection point was around 20 µmol/L, while the predicted concentration of ^•^OH from the density of DMPO at the inflection point was 125 µm^−1^, which corresponded to around 3.2 mmol/L. This shows that the ^•^OH generated by low LET radiation were localized. Therefore, the localized concentration of ^•^OH in a single spur might be higher than 3.2 mmol/L. The molecule-to-molecule distance of ^•^OH at a concentration of 3.2 mmol/L was estimated as 8 nm, as shown in Table 1. Figures 1 and 2 show that the position of the inflection point and the two types of liner trends were the same in all the experiments performed with different parameters. Thus, the density of ^•^OH in the spur volume was not affected by dose, dose rate, or ray type.

Caffeine competes with DMPO to scavenge ^•^OH in the reaction mixture. The 3.2 mmol/L ^•^OH could be canceled by 5 mmol/L caffeine, while 1.7 mol/L ^•^OH could not be canceled by 5 mmol/L caffeine. Figure 3 shows that only mmol/L-level ^•^OH was canceled using 5 mmol/L caffeine. However, a linear increase in DMPO-OH concentration, which implies extremely high ^•^OH generation in very close molecular proximity, was observed in Fig. 3. Thus, it was validated that an extremely high concentration of ^•^OH generated, which could not be controlled by 5 mmol/L caffeine. The highest concentration of DMPO examined in this study was 1.7 mol/L, which corresponded to 1 nm molecule-to-molecule distance.

The existence of extremely “dense” generation of ^•^OH can be detected only with extremely high density of DMPO. In other words, it was suggested that mmol/L-order of ^•^OH scavenger had no effect on “dense” ^•^OH generation. Controlling the localized extremely dense ^•^OH generation, which is more than 1.7 mol/L, using an ^•^OH scavenger would be unrealistic. Therefore, instead of controlling the extremely dense ^•^OH generation, regulation of secondary ROS such as H_2_O_2_, O_2_^•−^, and HO_2_^•^, will be an effective action.

Since the relations between the density of DMPO and concentration of DMPO-OH showed a stable three-phase profile that was independent of dose and/or dose rate, it could be expected that ^•^OH generated in a localized area, such as a spur. Low LET radiation induces secondary or later generated electrons by sequential interactions with molecules or atoms in the irradiated sample, subsequently producing a primary cluster of reactive species in the spur by such a sequence of ionization and/or excitation. Multiple spurs can form and scatter along with radiation track to the released radiation energy. The distances between isolated spurs are sufficiently larger than the diameter of the spurs.

It is considered that the ionizations occurring in a spur are random. Considering this to be true, the concentration of ^•^OH generation in a single spur may be constant. Assuming a constant mmol/L concentration of ^•^OH generation in a single spur, the extremely dense ^•^OH generation may be derived from the additional bias of ^•^OH generation at an initial time scale in the overlapping of spurs. Such overlapping spurs may be caused by track-end electrons, with more than 10 spurs overlapping in the very limited volume when the extremely dense, mol/L-level ^•^OH portions were made. To achieve two strikingly different densities corresponding to mmol/L and mol/L concentrations of ^•^OH by means of the overlapping spurs, the spur-to-spur distance must decrease precipitously at the track-end. Therefore, it is natural to think that originally two densities, i.e., mmol/L and mol/L levels, of ^•^OH generations occurred in a spur. The underlying mechanisms of extremely dense ^•^OH generation still have not been elucidated.

A 0.66 MeV of a single γ-photon can emit from an excited nucleus accompanied with β^−^ disintegration of ^137^Cs into ^137^Ba. The number of γ-photon reached to 200 µl of DMPO aqueous sample, when the sample was irradiated with 32 Gy γ-ray. The 200 µl sample, which was placed at the bottom of a 1.5 ml polyethylene microtube, was considered as a sphere for simplification of calculation. Approximately 2.7 × 10^13^ photons could emit from the target during irradiation (4 min). Since approximately 40 µmol/L ^•^OH was generated by γ-ray irradiation in the 200 µl volume of the sample (Fig. 1D), almost 180 molecules of ^•^OH were generated by a γ-photon. X-ray may give a similar amount of ^•^OH. The number of ^•^OH generated by low LET radiation was much larger than the number of emitted photon from the sample. This suggests that localized dense ^•^OH generation could occur through the sequence of the ionizing process.

The dense ^•^OH portion makes chemical regulation of radio-biological effect difficult. It was reported in the previous paper that the ratio of sparse and dense ^•^OH components induced by X-ray irradiation to aqueous samples was as 50% of sparse ^•^OH component and 50% dense ^•^OH component. In this paper, γ-ray also showed similar ratio of sparse and dense ^•^OH generations (Fig. 1D). This is in other words that 50% of ^•^OH induced by low LET radiation is able to be regulated chemically. Another 50% of ^•^OH, i.e., effect of dense ^•^OH, might be regulated by next chemical target generated by sequential radical reactions, i.e., H_2_O_2_, O_2_^•−^, and/or HO_2_^•^. It was mentioned in a previous paper^(20)^ that multiple abilities of an anti-oxidative drug, such as ^•^OH suppression, O_2_^•−^ suppression, H_2_O_2_ suppression, and reducing oxidized molecules, are essential to regulate radiobiological effects efficiently. The result of this paper again gives attention to such multiple targets for the regulation of radio-biological effect.

Further, the two different densities of ^•^OH generation in an aqueous sample caused by a low LET radiation was observed by the EPR spin-trapping method with high reproducibility.^(15)^ The localized generation of ^•^OH with two different densities was not affected by dose, dose rate, and/or the energy of photon. The addition of 5 mmol/L caffeine as a ^•^OH scavenger to the reaction system could cancel the contribution of sparse (mmol/L-level) ^•^OH generation, leaving a trace of extremely dense (mol/L-level) generation of ^•^OH. The localized extremely dense generation of ^•^OH by low LET radiation was thus established.

## Figures and Tables

**Table 1:** Concentration of DMPO and the calculated probe-to-probe distance and probe density

DMPO concentration (mM)	Probe-to-probe distance* (nm)	Probe density** (µm^−1^)
4.9 × 10^−1^	15	67
9.6 × 10^−1^	12	83
1.7	10	100
2.3	9	111
3.2	8	125
7.7	6	167
2.6 × 10^1^	4	250
6.2 × 10^1^	3	333
2.1 × 10^2^	2	500
4.9 × 10^2^	1.5	667
9.6 × 10^2^	1.2	833
1.7 × 10^3^	1	1,000

*The probe-to-probe distance was calculated as length of an edge of a cubic volume, which was occupied by one molecule of DMPO. **The probe density was defined as the reciprocal of probe-to-probe distance.
